# Screening and identification of host proteins interacting with *Theileria annulata* cysteine proteinase (TaCP) by yeast-two-hybrid system

**DOI:** 10.1186/s13071-017-2421-0

**Published:** 2017-10-30

**Authors:** Shuaiyang Zhao, Guiquan Guan, Junlong Liu, Aihong Liu, Youquan Li, Hong Yin, Jianxun Luo

**Affiliations:** 1 State Key Laboratory of Veterinary Etiological Biology, Key Laboratory of Veterinary Parasitology of Gansu Province, Lanzhou Veterinary Research Institute, Chinese Academy of Agricultural Sciences, Xujiaping 1, Lanzhou, Gansu 730046 People’s Republic of China; 2 Jiangsu Co-innovation Center for Prevention and Control of Important Animal Infectious Diseases and Zoonoses, Yangzhou, 225009 People’s Republic of China

**Keywords:** *Theileria annulata*, Cysteine proteinases, Yeast two-hybrid, Interaction, Crbn, Ppp4C

## Abstract

**Background:**

*Theileria annulata* can infect monocytes/macrophages and B lymphocytes and causes severe lymphoproliferative disease in ruminants. Meanwhile, infection by *T. annulata* leads to the permanent proliferation of cell population through regulating signaling pathways of host cells. Cysteine proteinases (CPs) are one kind of protein hydrolase and usually play critical roles in parasite virulence, host invasion, nutrition and host immune response. However, the biological function of *T. annulata* CP (TaCP) is still unclear. In this study, a yeast-two-hybrid assay was performed to screen host proteins interacting with TaCP, to provide information to help our understanding of the molecular mechanisms between *T. annulata* and host cells.

**Methods:**

The cDNA from purified bovine B cells was inserted into pGADT7-SfiI vector (pGADT7-SfiI-BcDNA, Prey plasmid) for constructing the yeast two-hybrid cDNA library. TaCP was cloned into the pGBKT7 vector (pGBKT7-TaCP) and was considered as bait plasmid after evaluating the expression, auto-activation and toxicity tests in the yeast strain Y2HGold. The yeast two-hybrid screening was carried out via co-transforming bait and prey plasmids into yeast strain Y2HGold. Sequences of positive preys were analyzed using BLAST, Gene Ontology, UniProt and STRING.

**Results:**

Two host proteins, CRBN (*Bos taurus* cereblon transcript variant X2) and Ppp4C (*Bos indicus* protein phosphatase 4 catalytic subunit) were identified to interact with TaCP. The results of functional analysis showed that the two proteins were involved in many cellular processes, such as ubiquitylation regulation, microtubule organization, DNA repair, cell apoptosis and maturation of spliceosomal snRNPs.

**Conclusions:**

This study is the first to screen the host proteins of bovine B cells interacting with TaCP, and 2 proteins, CRBN and Ppp4C, were identified using yeast two-hybrid technique. The results of functional analysis suggest that the two proteins are involved in many cellular processes, such as ubiquitylation regulating, microtubule organization, DNA repair, cell apoptosis and maturation of spliceosomal snRNPs. The interaction with CRBN and Ppp4C indicate that TaCP possibly is involved in regulating signaling pathways and cell proliferation, which is helpful for understanding the interaction between *T. annulata* and host cells.

## Background

*Theileria annulata* is an intracellular protozoan parasite that can be transmitted by ticks of genus *Hyalomma*, such as *H. detritum* and *H. anatolicum anatolicum* [1]. As a malignant *Theileria* species causing tropical theileriosis in North Africa, Southern Europe, India, the Middle East and Asia [2], it can infect bovine monocytes/macrophages and B lymphocytes [3], and causes severe lymphoproliferative disease in ruminants [4]. *Theileria annulata*-infected cells can be cultured in vitro and characterized by permanent proliferation of cell populations. Meanwhile, the presence of *T. annulata* in bovine cells is essential for the transformed state, and this phenomenon is reversible and can be ceased by treating with the drug Buparvaquone 720c (BW720c) [5].

Cysteine proteinases (CPs) are a kind of protein hydrolase, with cysteine residues in its active center, and are also referred to cysteinyl peptidases [6]. In the MEROPS peptidase database (http://merops.sanger.ac.uk), CPs are classified into nine classes, referred to as clans (clan CA, CD, CE, CF, CH, CL, CM, CN and CO, and one unclassified clan) [6, 7]. In Apicomplexan parasites, CPs play critical roles in parasite virulence, host invasion, nutrition and host immune response [8, 9]. Currently, the study of CPs from apicomplexan parasites focuses mainly on using the inhibitors for treatment of parasitic diseases. For instance, Nene et al. [10] found a diazomethyl ketone inhibitor of CPs impaired the growth of *T. parva-*infected lymphocytes; and Holman et al. [11] showed CP inhibitors E-64d reduced the *T. equi* propagation; Okubo et al. [12] proved the addition of CPs inhibitors, E-64d and ALLN reduced the growth of *Babesia bovis* and the parasite invasion. However, studies identifying CP interaction proteins, and exploring its function in regulating signaling pathways of host cells, are rare.

Without the restriction of a parasitophorous vacuole, membrane or secreted proteins from *T. annulata* have the opportunity to interfere with host cell signaling pathways that regulate cell proliferation and survival [13]. To uncover the mechanism of transformation, many recent studies have focused on the identification of *T. annulata* proteins that interact with bovine lymphocytes tranformation. For example, it was proved that *T. annulata* secretory protein (TaSE) expressed during schizont and piroplasma stages interacted with α-tubulin of bovine cells by immunoprecipitation (IP) and potentially involved into mitosis [14]. The overexpression of gp34, a GPI-anchored protein on the surface of *T. annulata* schizonts, could induce cytokinetic defects and resulted in accumulation of binucleated cells, suggesting that gp34 participates in regulating host cell division [15]. The members TashAT family, *TashAT1*, *TashAT2*, *TashAT3* and *SuAT1* were found to harbor 4, 3, 4 and 1 AT hook DNA-binding domains, respectively. They can be transported to nucleus of *T. annulata*-infected cells and interfer with host cell proliferation [16–18]. Based on the results of co-IP and pull-down experiments, Seitzer et al. [19] found that *T. annulata* surface protein, TaSP, interacted with the host cell microtubule network, such as spindle poles, mitotic spindle apparatus and mid-body during host cell mitosis, which indicates that TaSP play a role in the parasite distribution into daughter host cells. Another *T. annulata* membrane protein, P104, is a polymorphic protein on the schizont surface. Woods et al. [20] demonstrated that it can strictly recruit end-binding protien1 (EB1) to regulate host cell microtubule network dynamics through a SxIP motif of P104. Marsolier et al. [21] found a prolyl-isomerase of *T. annulata*, TaPIN1, that can interact with the host ubiquitin ligase FBW7 and promot host cell transformation through stabilization of c-JUN. In addition, the relationships of sub-telomere-encoded variable secreted protein (SVSP) and HSP90 with lymphocyte transformation due to *Theileria* infection have also been described [22, 23]. To date, most reports have indicated that the presence of lymphocyte transformation due to *T. annulata* infection is involved in regulating signaling pathways of host cells, such as NF-κB, c-Jun NH_2_-terminal kinase (JNK), phosphoinositide-3 kinase (PI3-K) kinase B, protein kinase-A (PKA), Notch and c-Myc pathways [24–29]. However, the mechanism of *T. annulata* proteins regulating signaling pathways of host cells are mostly unknown. Recently, some putative membrane or secreted proteins were screened out based on genomic data, which might be relevant to the transformation of *Theileria* infected cells [21, 30]. As a protein family, CPs of *T. annulata* have many members, based on the complete genome sequences of *T. annulata* [30], and some CP genes with high homology in different genera may have similar function. Through comparative genomics with that of *T. parva*, Pain et al. [30] found that one CP gene from *T. annulata* (accession no.: XM_947478.1), TaCP, might be involving in regulating cell proliferation. The analysis results in the MEROPS peptidase database showed that TaCP was classified as family C1, sub-family C1A (papain family, clan CA), and had no cysteine residue in the catalytic center and classed as non-peptidase homologues, which had been shown experimentally to lack peptidase activity or lack one or more of the active site residues. Research on TaCP will provide useful information regarding the function of this kind of CP in different parasites, which had no cysteine residue in the catalytic center. In this study, to better understand the interaction between *T. annulata* and host cells, the TaCP was used as bait plasmid to select host proteins from a cDNA library of bovine B cells by yeast-two-hybrid system.

## Methods

### Cell culture

The *T. annulata* **(**Neimeng1) schizont-infected cell line was obtained and conserved by the Vector and Vector-borne Disease (VVBD) laboratory, Lanzhou Veterinary Research Institute (LVRI), China. Cells were cultured using RPMI 1640 (Gibco, Grand Island, New York, USA) supplemented with 10% fetal calf serum (Gibco, Grand Island, New York, USA) and 100 mg/ml penicillin/streptomycin in a humidified 5% CO_2_ atmosphere at 37 °C.

### Construction of yeast two-hybrid cDNA library of bovine B cells

Peripheral blood was collected in 9 ml sterile K3EDTA vacutainers from 18 month-old piroplasma-free cattle. Peripheral blood mononuclear cells (PBMCs) were isolated using a bovine PBMC separation medium kit (Haoyang Biotec, Tianjin, China) through density gradient centrifugation. B cells were separated from PBMCs according to the manual of Anti-PE MicroBeads (Miltenyi Biotec, Bergisch Gladbach, Germany). Briefly, 10^8^ of PBMCs was used, labeled with PE-conjugated mouse anti-bovine CD21 McAb (Bio-Rad, Hercules, California, USA) and then incubated with anti-PE MicroBeads after flow cytometer analysis. The magnetically labeled B cells were purified with a MACS separator and analyzed with flow cytometry. Finally, the purified B cells were sent to Takara (Dalian, China) for construction of yeast two-hybrid cDNA library. The total RNA of B cells was extracted and reverse transcribed into 1st strand cDNA. Following the normalization treatment and short-fragment removal, the cDNA of bovine B cells was cloned into pGADT7-SfiI vectors (Prey Plasmid), which had three different reading frames to confirm the right expression of every protein.

### Bait plasmid construction

Total RNA of *T. annulata* infected cells was extracted by using TRIzol Reagent following the manufacturer’s protocol (Thermo Fisher, Waltham, Massachusetts, USA), and the cDNA was obtained through reverse transcription by using a 1st strand cDNA Synthesis kit (Takara, Dalian, China). Referring to the sequence of TaCP in GenBank (accession no.: XM_947478.1), the TaCP gene fragment encoding 348 residue (from aa66 to aa413) peptide (Fig. 2a), was amplified by PCR from the cDNA, based on the specific primers (restriction site underlined): TaCP-F (5′-CGC GGA TCC GTT CAT CAG GCA GAA GCG CAA TC-3′); TaCP-R (5′-AAC TGC AGT ACT GCG TAT ACT GCA AAG-3′). Subsequently, the PCR product was purified, digested with restriction enzyme *BamH I* and *Pst I* (NEB, Beverly, Massachusetts, USA), and inserted into pGBKT7 plasmid. The recombinant pGBKT7- TaCP plasmid (bait plasmid) was confirmed by double restriction enzyme digestion and sequencing (Sangon Biotech, Shanghai, China).

### Bait plasmid expression in yeast cells

Following the manufactures protocols of the Yeastmaker™ Yeast Transformation System 2 kit (Cat. No. 630439, Clontech, Mountain View, California, USA), the recombinant pGBKT7- TaCP plasmid was transformed into yeast strain Y2HGold, and the transformants were screened on agar plates containing the minimal yeast medium without tryptophan (SD/-Trp) at 30 °C for 3–5 days. To check the expression of TaCP bait in Y2HGold, one colony from the SD/-Trp plate was incubated into SD/-Trp broth and grown to 0.6 of OD600 at 30 °C, 250 rpm. Subsequently, total proteins were extracted from the centrifuged pelleted cells by the Urea/SDS method [31]. The extracted proteins were separated by 12% SDS-PAGE and electro-blotted onto PVDF membrane (Millipore, Billerica, Massachusetts, USA) for western blot analysis. The CP bait expression was detected with anti-Myc tag mouse McAb (Cat. No. 66004-1, Proteintech, Rocky Hill, New Jersey, USA), followed by peroxidase-conjugated goat anti-mouse secondary antibody (Cat. No.A0168, Sigma, Saint Louis, Missouri, USA), with positive signals revealed using the 5-bromo-4-chloro-3-indolyl phosphate/nitro blue tetrazolium (BCIP/NBT) liquid substrate system (B1911, Sigma, Saint Louis, Missouri, USA). The transformants of pGBKT7-53 plasmid were used as a positive control.

### Auto-activation and toxicity tests of bait plasmid

Following the manufactures protocols of the Matchmaker™ Gold Yeast Two-Hybrid System (Clontech, Mountain View, California, USA), the pGBKT7- TaCP and pGBKT7 plasmids were transformed into Y2HGold, respectively. Transformants were grown on SD/-Trp, SD/-Trp/X (40 μg/ml X-α-Gal) or SD/-Trp/X /A (40 μg/ml X-α-Gal and 125 ng/ml Aureobasidin A) agar plates at 30 °C for 3–5 days. Followed by white or very pale blue colonies on SD/−Trp and SD/-Trp/X plates, and absence of colony growth on SD/-Trp/X/A plates, the bait was confirmed without auto-activation. If the bait was toxic, the colonies containing the bait plasmid were significantly smaller than colonies containing the pGBKT7 plasmid. Only the bait plasmid without auto-activation and toxicity could be used in yeast-two-hybrid screening.

### Yeast-two-hybrid screen using co-transformation of bait with prey plasmids

In order to screen host proteins that interact with TaCP bait against yeast two-hybrid, a cDNA library of purified bovine B cells, pGBKT7- TaCP and prey plasmids were co-transformed into Y2HGold with Yeastmaker™ Yeast Transformation System 2 according to the manufactures protocols. Briefly, the pGBKT7- TaCP and prey plasmids were co-transformed into Y2HGold, and the co-transformants were then grown on SD/-Leu/-Trp/ X-α-Gal/AbA (DDO/X/A) agar plates at 30 °C for 3–5 days. Blue colonies were patched out onto higher stringency SD/-Ade/-His/-Leu/-Trp/X-α-Gal/AbA (QDO/X/A) agar plates. To estimate each insert size of potential positive prey plasmids, primers of pGADT7-F/R, sequence information was provided by Takara, were used in PCR amplification.

### Confirmation of the interactions

To confirm the interactions, co-transformations of pGBKT7- TaCP bait into Y2HGold with each prey plasmid in putatively positive hits were carried out. Briefly, the prey plasmids were extracted from putatively positive clones using the Easy Yeast Plasmid Isolation Kit (Cat. No. 630467, Clontech, Mountain View, California, USA). Subsequently, each prey plasmid was transformed into *E. coli* DH5α competent cells (Transgen, Beijing, China), and purified from transformants growing on selected LB/Amp agar plates by using the Plasmid Mini Kit I (Cat. No. D6943-02, Omega, Doraville, Georgia, USA). Following this, each putatively positive prey plasmid was co-transformed with pGBKT7- TaCP bait and pGBKT7 plasmids into Y2HGold and the co-transformants grown on QDO/X/A plates to test for interactions. Co-transformant containing pGADT7-T and pGBKT7-Lam, grown on QDO/X/A plates, was used as a negative control and co-tansformant containing pGADT7-T and pGBKT7-53, grown on QDO/X/A, was used as a positive control. True positive interactions were indicated by blue colonies under these conditions.

### Positive prey analysis

Positive prey plasmids were sequenced and the results blasted against NCBI databases to analyze the function of the corresponding bovine genes. Protein function of the identified genes was analyzed using Gene Ontology (http://amigo.geneontology.org/amigo), UniProt database (http://www.uniprot.org/), and STRING (http://string.embl-heidelberg.de/).

## Results

### Construction of yeast two-hybrid cDNA library of bovine B cells

**Fig. 1:**
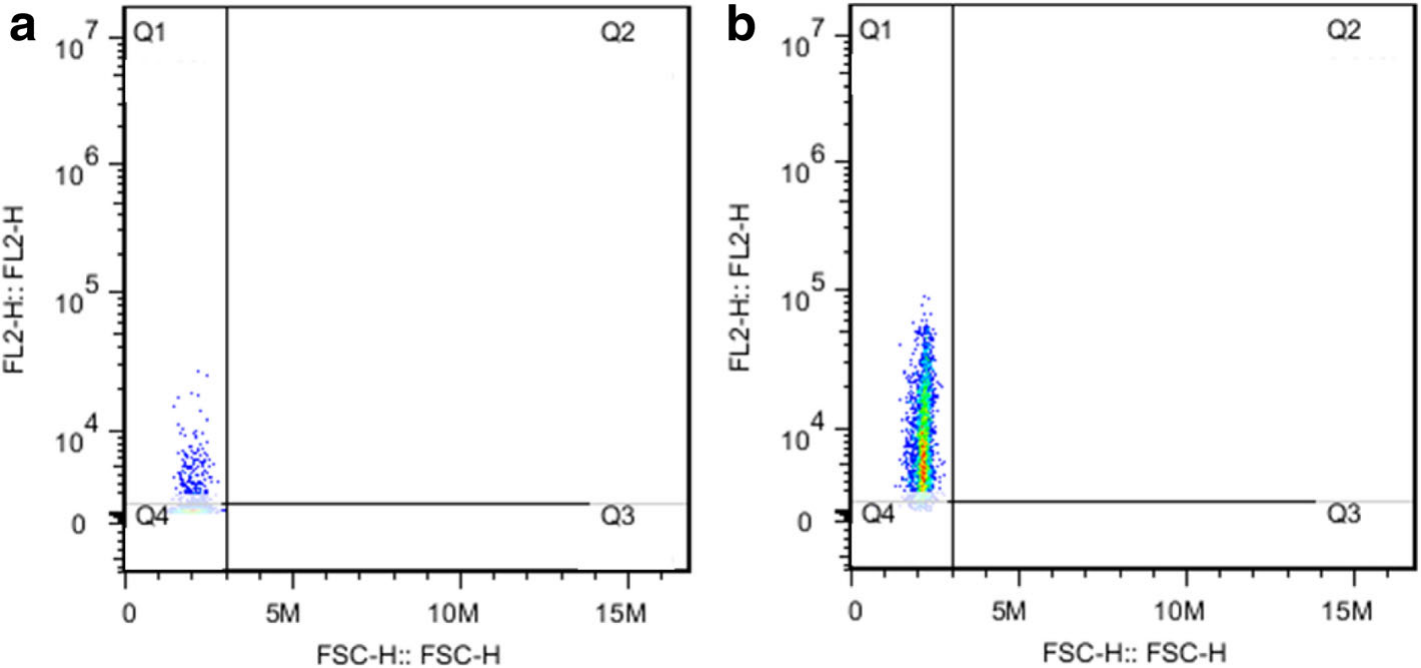
Identification of bovine B cell purity by flow cytometry. **a** Analysis of B cell proportion in PBMCs, 21.8% of B cells in bovine PBMCs. **b** Purity analysis of B cells separated with Anti-PE MicroBeads, purity = 95.3%

Following the method of density gradient centrifugation, PBMCs were separated from EDTA-anticoagulant bovine peripheral blood. After incubation with primary antibody of PE-conjugated mouse anti-bovine CD21 monoclonal antibody (McAb), PBMCs were analyzed by flow cytometry; the purity of B cell in PBMCs was identified as 21.8% (Fig. 1a). Subsequently, B cells were separated from the PBMCs using anti-PE MicroBeads; the purity of B cells was identified as 95.3% (Fig. 1b), which satisfied the purity requirement of cDNA library construction of B cells. 2 × 10^7^ purified B cells were obtained and sent to Takara for cDNA library construction. The quality report of cDNA library showed the cDNA library titer was 2 × 10^6^ cfu, and the size of inserted fragments was from 750 to 3500 bp, according to the sequencing results of 96 clones. These results indicated that the cDNA library could be used for yeast-two-hybrid screening. In addition, the prey plasmid provided by Takara was 1 mg/ml in concentration.

### Construction of pGBKT7- TaCP bait plasmid and TaCP expression

**Fig. 2:**
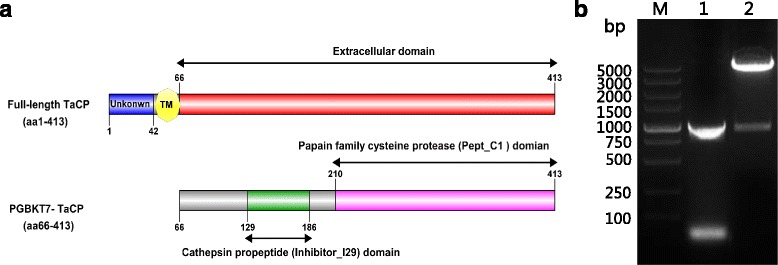
Gene structure and bait plasmid construction of TaCP. **a** Gene structure of TaCP and the region (aa66-413) of TaCP used in yeast-two-hybrid screening. **b** Construction of pGBKT7-TaCP bait plasmid. Lane M: DL5000 DNA Marker; Lane 1: amplified fragment of TaCP from *T. annulata* cDNA; Lane 2: confirmation of pGBKT7-TaCP by digestion with *BamH I* and *Pst I*

**Fig. 3:**
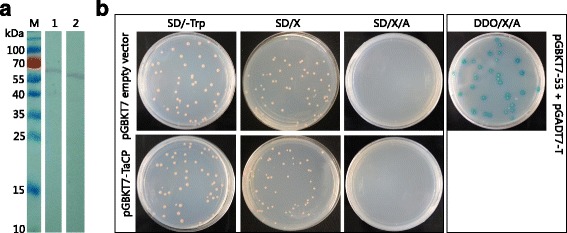
Expression, auto-activation and toxicity tests for pGBKT7-TaCP bait. **a** Western blot analysis of total protein extracts of Y2HGold. Lane 1: pGBKT7-TaCP; Lane 2: pGBKT7-53. **b** Determination of the auto-activation and toxicity activity of the pGBKT7-TaCP bait plasmid in yeast cells. The pGBKT7-TaCP bait and pGBKT7 plasmids were used to transform Y2HGold cells and then grown on different plates. The co-transformants containing pGADT7-T and pGBKT7-53 were grown on DDO/X/A plates as positive control

The TaCP gene fragment encoding extracellular domain was amplified from cDNA of *T. annulata-*infected cells, at a size of 1044 bp. The construction of pGBKT7- TaCP bait plasmid was confirmed successfully by restriction enzyme digestion analysis (Fig. 2b). To detect the bait expression in Y2HGold before yeast-two-hybrid screening, total proteins of the Y2HGold transformed with bait plasmid were extracted and detected by western blot using anti-Myc tag mouse McAb. The molecular weight of TaCP bait was 60 kDa, which was consistent with its calculated size (Fig. 3a). As a positive control, the pGBKT7-53 vector transformed Y2HGold expressed a 57 kDa protein (Fig. 3a).

### Auto-activation and toxicity tests of the bait plasmid

To test the auto-activation activity of the bait protein in yeast cells, the pGBKT7 and pGBKT7-TaCP bait plasmids were transformed into Y2HGold and subsequently the transformants were grown on SD/-Trp, SD/-Trp/X and SD/-Trp/X /AbA plates (Fig. 3b). As a positive control, transformants containing pGADT7-T and pGBKT7-53 were grown on DDO/X/A plates. The colonies containing pGBKT7 or pGBKT7-TaCP bait plasmids were white in color on SD/-Trp/X plates, and as for the positive control, the colonies were blue on DDO/X/A plates, which indicated pGBKT7-TaCP bait without auto-activation activity (Fig. 3b). The colony size of Y2HGold transformed with bait plasmid was similar to that of Y2HGold transformed with the pGBKT7. Based on these results, the pGBKT7-TaCP bait plasmid could be used in the yeast-two-hybrid screening.

### Yeast-two-hybrid screening and confirmation of the interactions

**Fig. 4:**
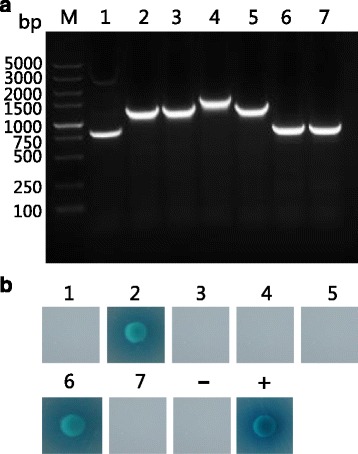
Analysis of putatively positive colonies. **a** Agarose gel electrophoresis analysis of amplified PCR products of the inserts on putatively positive prey plasmids. Lane M: DNA 5000 maker; Lanes 1–7: PCR amplification products of the inserts on the seven putatively positive hits. **b** Confirmation of putative hits. Y2HGold cells were co-transformed with pGBKT7-TaCP and each of the seven putatively positive prey plasmids (numbers 1–7) were plated on QDO/X/A plates; positive interaction was indicated by the presence of blue colonies. Co-transformation with pGADT7-T and pGBKT7-Lam was used as a negative control, while co-transformation with pGADT7-T and pGBKT7-53 was used as a positive control

After co-transformation of pGBKT7-TaCP and prey plasmids and growth on DDO/X/A plates, 37 blue clones were obtained. Subsequently, these 37 blue clones were patched out onto higher stringency QDO/X/A plates. Seven of the 37 colonies still manifested blue color, indicating that they were likely to be positive hits. These seven prey plasmids were then isolated from their corresponding colonies and rescued through transformation of *E. coli* DH5α cells. After PCR amplification using the primers of pGADT7-F/R, the size of inserted fragment in each prey plasmid was shown by gel electrophoresis (Fig. 4a)*.* To eliminate false positive hits, each of the seven prey plasmids was co-transformed with pGBKT7-TaCP into Y2HGold cells and the co-transformants cultivated on QDO/X/A plates. The blue clones observed on QDO/X/A plates formed two of the seven co-transformants (Fig. 4b). Meanwhile, each of the seven prey plasmids was co-transformed with pGKBT7 plasmid into Y2HGold cells, with no colony growth on QDO/X/A plates (data not shown). Based on the above results, two host proteins were identified to interact with TaCP.

### Sequencing and analysis of positive prey

To confirm nucleotide sequence information of the identified host proteins interacting with TaCP, the two prey plasmids were sequenced using the primers of pGADT7-F/R. The sequences were analyzed using the BLAST tool in NCBI. The two fragments had 99% similarity with *Bos taurus* cereblon transcript variant X2 (CRBN, XM_015459512.1) and *Bos indicus* protein phosphatase 4 catalytic subunit (Ppp4C, XM_019988010.1). The 2 sequences encoded each 177 and 125-residue fragments with C terminus of CRBN and Ppp4C. Gene ontology, UniProt and STRING analysis showed CRBN was a substrate recognition component of a DCX (DDB1-CUL4-X-box) E3 protein ligase complex that mediates the ubiquitination and subsequent proteasomal degradation of target proteins, and was required for limb outgrowth and expression of the fibroblast growth factor FGF8 as a substrate receptor. Ppp4C catalyzed the hydrolysis of various bonds, such as C-O, C-N, C-C, phosphoric anhydride bonds and was involved in many processes, such as microtubule organization at centrosomes, maturation of spliceosomal snRNPs, apoptosis, DNA repair, tumor necrosis factor (TNF)-alpha signaling. The Ppp4C-Ppp4R1 PP4 complex may play a role in dephosphorylation and regulation of HDAC3 (Table 1).

**Table 1:** Analysis results of the two prey proteins based on BLAST, Gene ontology, UniProt and STRING databases

Protein	Accession No.	Function annotation
*Bos taurus* cereblon transcript variant X2 (CRBN)	XM_015459512.1	A substrate receptor of E3 ubiquitin ligase complex, mediates the ubiquitination and degradation of target proteins, involves in learning and memory by regulating the large-conductance calcium-activated potassium channels in brain regions
*Bos indicus* protein phosphatase 4 catalytic subunit (Ppp4C)	XM_019988010.1	The catalytic subunit of ubiquitous protein serine/threonine phosphatase 4, involves in microtubule organization, maturation of spliceosomal snRNPs, apoptosis, DNA repair, TNF-α signaling, activation of JNK, regulation of histone acetylation, DNA damage checkpoint signaling, NF-κB activation and cell migration

## Discussion

CPs are referred to thiol-peptidases, sulfhydryl peptidases or cysteinyl peptidases, and play important roles in parasite virulence, host invasion, nutrition and host immune response [6, 8, 9]. In general, CPs are classified into nine classes, such as clan CA and clan CD. To date, 84% of the known parasite cysteine peptidase sequences belong to 12 families, including peptidases in clan CA [6]. In the MEROPS peptidase database, TaCP was classified as family C1, sub-family C1A (papain family, clan CA). Many members of sub-family C1A were endopeptidases and often part of host-pathogen interactions. Moreover, they were also utilized for host invasion in many parasites [32]. Thus, functional studies of CP in *T. annulata* are necessary for better understanding the interaction between *T. annulata* and host cells. In the study, bovine B cells were separated from PBMCs using magnetic microbeads and its purity was identified as 95.3% by flow cytometry. Subsequently, a yeast two-hybrid cDNA library was constructed using the purified bovine B cells. It had 2 × 10^6^ cfu of titer and 750–3500 bp of inserted fragment size, which indicated the library could be used in yeast two-hybrid screening. Structure analysis of TaCP using online software SMART showed that it contained a transmembrane domain (aa42-65) and two functional domains, cathepsin propeptide (Inhibitor-129) domain (aa129-186) and papain family cysteine (Pept-C1) domain (aa210-413). And the 1044 bp fragment encoding a 348-resiude (aa66-413) peptide of TaCP, which included two functional domains, was clone into pGBKT7 vector as the bait plasmid to screen host proteins interacting with TaCP by yeast-two-hybrid system. Finally, two host proteins, CRBN and Ppp4C were indicated to interact with TaCP. It was the first report that CRBN and Ppp4C interacted with TaCP. CRBN (Cereblon), a substrate receptor of E3 ubiquitin ligase complex, is highly conserved in animals and plants and is located in cytoplasm, nucleus and peripheral membrane [33–38]. Higgins et al. [39] found that wild-type CRBN protein was important for memory and learning, and the C-terminal 24 amino acids were critical for CRBN function. Lee et al. [38] used yeast-two-hybrid system to screen CRBN partners from rat brain cDNA. The results showed rat CRBN directly and specifically interacted with the α1 subunit of AMPK (adenosine monophosphate-activated protein kinase), combined with CO-IP and pull down experiments, which indicated CRBN plays a potential role in energy-balance by interacting with AMPK. Using CRBN knockout mice, Lee et al. [40] found mouse embryonic fibroblast cells from CRBN-deficient mice were strongly resistant to ER stress-mediated cell death, indicating the role of CRBN in cellular stress signaling. Together with damaged DNA-binding protein 1, cullin-4A/B and regulator of cullins 1, CRBN forms a Cullin 4-RING E3 ubiquitin ligase (CRL4^CRBN^) complex, which promotes the ubiquitination of many proteins [41]. According to the roles of CRBN in host cells, *T. annulata* may regulate CRBN dependent processes and play similar roles in host cells through TaCP-CRBN interaction, which deserves further investigation.

Another protein identified, Ppp4C, the catalytic subunit of ubiquitous protein serine/threonine phosphatase 4 (Ppp4), is highly conserved between invertebrates and vertebrates [42]. Helps et al. [43] and Sumiyoshi et al. [44] found the decrease of Ppp4c in *Drosophila* and *C. elegans*, respectively, reduced the viability of embryos, which indicated Ppp4c to be essential in the processes of nucleation, growth and organization of microtubules, formation of the mitotic spindle. Voss et al. [45] found Ppp4c regulates microtubule organization at centrosomes during cell division when cells were treated with spindle toxins, nocodazole and paclitaxel, indicating Ppp4c to play an important role in cell mitosis. To date, it has been reported that Ppp4c was involved in regulate various signaling pathways through dephosphorylating the downstream molecular, such as NF-κB pathway, c-Jun N-terminal kinase (JNK) activation, Smoothened (Smo)-mediated Hedgehog signaling and apoptosis regulator PEA-15 [46–49]. Based on these studies, Ppp4c may play an important role in cell proliferation. However, whether Ppp4c can regulate the proliferation of *T. annulata* infected cells or not still needs further study.

In this study, the cDNA of bovine B cells was cloned into pGADT7-SfiI vectors, which had three different reading frames to ensure the right expression of every protein. Following a high throughput yeast-two-hybrid screening, 7 putative positive clones were obtained. However, only two host proteins were identified to interact with TaCP after a co-transformation experiment of pGBKT7-TaCP with each of the 7 prey plasmids, which indicated this assay had a high false positive rate. Using co-transformation of bait with prey plasmids to screen host proteins also has lower efficiency than that of yeast mating assay. Meanwhile, the screening experiment was only taken one time, so some interactive host proteins are missed from our screen, especially for some weakly interacting ones. Because of these limitations, it is possible that there are other TaCP binding partners. In the future, new interacting host proteins need to be identified, and further study on CRBN and Ppp4C also needs to be carried to help understand the interaction between *T. annulata* and host cells.

## Conclusions

In the present study, TaCP was used as bait to screen its interacting host proteins from bovine B cell cDNA library, and two host proteins, CRBN and Ppp4C, were screened out and identified. It was the first report of CRBN and Ppp4C interaction with TaCP*.* According the functional analysis of CRBN and Ppp4C, we found that these two host proteins to be involved in many cellular processes, such as ubiquitylation regulating, microtubule organization, DNA repair, cell apoptosis and maturation of spliceosomal snRNPs. The results suggest TaCP may be involved in regulating signaling pathways and cell proliferation, which helps us better understand the interaction between *T. annulata* and host cells. In the future, further study should be focused on TaCPs, including the confirmation of functional peptide, modification of key amino acid site, the identification of the regulated signaling pathway and the effect on host cell proliferation.

## Abbreviations

-Ade: Without adenine
Amp: Ampicillin
AMPK: Adenosine monophosphate-activated protein kinase
BW720c: Buparvaquone 720c
CRBN: Cereblon
-His: Without histidine
IFA: Indirect immunofluorescence assay
IP: Immunoprecipitation
-Leu: Without leucine
McAb: Monoclonal antibody
Ppp4C: Protein phosphatase 4 catalytic subunit
TaCP: *Theileria annulata* cysteine proteinase
-Trp: Without tryptophane
